# 

**Published:** 1852-07

**Authors:** 


### CONTENTS.

ORIGINAL COMMUNICATIONS:— PAGE.

ART. 1.—On the Pathology of Spontaneous Hœmorrhage, by A. B. Palmer, M. D., - - - - - - - - - - - - - - 97
ART. 2.—Cases illustrative of the effects of an impaired condition of the blood upon the general system, by U. P. Golliday, M. D., - - - - 103
ART. 3.—On the Treatment of Dysentery, by C. W. Davis, M. D., - 108
ART. 4.– Case of Spina Bifida, by J. P. Russell, M.D., - - - - 109
ART. 5.—Singular Effects of a Hœmorrhagic Diathesis, by N. S. Davis, M. D., - - - - - - - - - - - - - - 110
ART. 6.—Case of Monstrosity, by M. Miles, M. D., - - - - 111

SELECTIONS:—

Neuralgia Treated by Black Snake Root, - - - - - - - 113
On the Reparative Power of the Spinal Cord after complete division, - 117
Microscopic Observations respecting Arterial and Capillary Circulation, 120
Influence of the Imagination or the Will upon the Pregnant Woman, - 123
Nitric Acid in Hooping Cough and Asthma, - - - - - - 125
On the Treatment of Pneumonia by the inhalation of Chloroform, - - 126
On Iodine Clysters in the Treatment of Dysentery, - - - - - 127
Sesquicarbonate of Ammonia in Lepra and Psoriasis, - - - - - 127
Collodion in Erysipelas, - - - - - - - - - - - 128
Bella donna as a Palliative in Epilepsy, - - - - - - - - 128

EDITORIAL:—

Notice of Wood's Practice of Medicine, - - - - - - - 129
Meig's Velpeau's Midwifery, - - - - - - - - - - 130
Report of the Eastern Lunatic Asylum, Williamsburg, Va., &c., - - 130
What Foreigners think of us, - - - - - - - - - - - 131
Pneumonia, - - - - - - - - - - - - - 134
Cook Co. Medical Society, - - - - - - - - - - 137
Knox Co. Medical Society, - - - - - - - - - - 143

